# Depression and anxiety: maladaptive byproducts of adaptive mechanisms

**DOI:** 10.1093/emph/eow019

**Published:** 2016-07-04

**Authors:** Carl T. Bergstrom, Frazer Meacham

**Affiliations:** 1 Department of Biology, University of Washington, Seattle, WA 98125, USA

Depression and anxiety disorders inflict untold harm on an enormous number of people. In the United States in a single year, nearly 10% of the population will suffer from a mood disorder and more than 20% will suffer from an anxiety disorder. Over the course of a lifetime, these numbers increase to 20% for mood disorders and 30% for anxiety disorders (National Institute of Mental Health 2016). From an evolutionary perspective, the prevalence of depression and anxiety disorders poses a serious puzzle. The typical onset of these disorders occurs before or during an individual’s reproductive years (Kessler et al., 2005) and they can be severely detrimental to even basic daily functioning. Why has natural selection left us vulnerable in this way?

The inaugural George Williams Prize has been awarded to the authors of a paper that takes an important step toward answering this question (Trimmer et al., 2015). Our aim here is to outline their novel approach, explain how they use it to make sense of low mood and depression, and illustrate its breadth and power by considering an application to pathological anxiety.

Within an evolutionary medicine framework, one might attempt to explain mental illness in a number of ways. A null hypothesis for any such disorder might be that disease cases represent the maladaptive extremes of the population distribution of a complex behavioral trait, determined by the interaction of genetic and environmental variation (Stearns and Medzhitov, 2016). This null hypothesis is probably underemployed in evolutionary explanations of uncommon pathologies. However, it seems insufficient to explain disorders as common as depression and anxiety. At the other end of the adaptationism spectrum, some evolutionary psychologists have postulated that common mental illnesses are useful. By this view, depression is not a disorder at all, but rather an adaptation for bargaining, conflict avoidance, problem-solving, disease avoidance, or other purposes (e.g. Hagen 2003; Price et al. 1994; Andrews and Thomson 2009; Anders et al. 2013). Some of these may be reasonable hypotheses for the evolutionary role of ordinary low mood, but none seem adequate to explain the severe and prolonged symptoms associated with clinical depression (Nettle, 2004).

Trimmer and colleagues advocate a more nuanced view: while the behaviors associated with mood disorders and anxiety disorders are not themselves adaptive, but may arise from adaptive mechanisms that have become dysregulated by the stochastic inputs they receive. We concur. Depression appears to be an extreme and persistent form of ordinary low mood, clinical anxiety an extreme and often persistent form of justified anxiety. To explain depression and anxiety disorders, then, we need to do two things. First, we must understand the adaptive significance of these mental states when they are functioning properly (Nesse, 1990). Second, we need to explain why they are prone to malfunction.

Over the past 15 years, researchers have made substantial progress toward the first of these goals by viewing mood and anxiety as evolved mechanisms that modulate behavior. Anxiety increases sensitivity to signs of potential threat, preparing an individual to respond appropriately to dangerous circumstances (Nesse, 2001; Bateson et al., 2011). Even if danger is rare it may be beneficial to experience anxiety frequently, just as a well-tuned smoke detector may generate numerous false alarms for each actual fire. Because the costs of failing to detect actual dangers far outweigh the costs of being unnecessarily anxious, an optimized anxiety response may trigger numerous false alarms for every true threat (Nesse, 2005). The benefit of low mood is not quite as obvious; its main behavioral effect is to decrease an individual’s motivation and activity. This can be advantageous at times when activity would be pointless, too energetically expensive, or excessively dangerous (Nettle and Bateson, 2012).

After these explanations of normal mood and anxiety, we still need to explain why these systems are prone to dysregulation and the associated mental illnesses. Evolutionary mismatch (Williams and Nesse, 1991) is an obvious candidate. Many aspects of our current social and ecological circumstances differ radically from the rest of our evolutionary history; it would be unsurprising if some of these evolved mechanisms were no longer optimal today. For example, if mood is involved in modulating goal pursuit and we now strive for longer-term goals and face more protracted periods of failure than we did in the past, an evolutionary mismatch could leave us prone to pathological depression when goals remain unmet for extended periods (Klinger, 1975; Nesse, 2009).

Instead of considering environmental mismatch or looking for ways in which mental illness is adaptive, Trimmer and colleagues model a situation in which an individual needs to regulate its activity in response to information it gets from the environment (Trimmer et al., 2015). After determining the individual’s optimal strategy, their approach is to ask whether this strategy sometimes produces instances of behavior that appear maladaptive when considered in isolation. If so, selection will not eliminate such behaviors, because they arise as byproducts of the strategy that is optimal overall. Any alternative strategy that avoids these particular mistakes will necessarily perform less well overall.

In the Trimmer et al. model, an agent faces a series of opportunities which it can either pursue or decline. The environment may be propitious, in which case expending the effort required to pursue an opportunity is likely to pay off, or the environment may be unfavorable, in which case the likelihood of failure is high and the agent does better declining. The only way for the agent to learn whether things are favorable or not is through trial and error. When recent efforts have been successful, it is best to continue to be active, and when recent efforts have failed, it is best to stop pursuing the opportunities—at least temporarily. Although recent experience is usually a good guide, the results of pursuing an opportunity are stochastic: sometimes an individual will succeed in an unfavorable environment or fail in a favorable one. Thus, it is possible for the agent to be misled by an unlikely sequence of successes or failures. And because the prudent response to failure is to stop trying, individuals who are unlucky enough to fail in a propitious environment are likely to stop trying and thus not discover that they were merely unlucky. In this way, maladaptive inactivity can arise in a subset of the population even when everyone is following an optimal behavioral rule.

**Figure 1.**
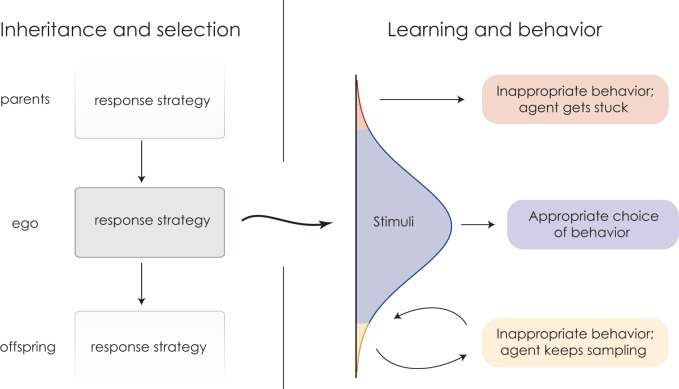
**Mistaken inferences are inevitable in stochastic environments**. Strategies for responding to experience are transmitted from parent to offspring (left side); it is these strategies that evolve. But the behaviors that individuals express arise through the interactions between response strategies and stochastic environmental stimuli (right side). Some behavior will end up being sub-optimal because by chance some stimuli will be misleading. Working within this framework, Trimmer et al. show that even with the optimal response strategy, some individuals will undergo uncharacteristic experiences and thus behave inappropriately given circumstances. Moreover, some of these individuals may retreat from further activity and thereby fail to gain additional information that could correct their misperceptions.

Viewing depression within this framework provides a new answer to the question of why depression persists evolutionarily. The trait that evolves is the strategy for responding to the entire range of possible experiences, but when we observe depression we are seeing only one particular behavior arising from the interaction of a response strategy and a particular set of environmental stimuli and experiences (Fig. 1). Natural selection can at best shape responsive behavior to maximize average payoff; if an individual receives atypical stimuli, the resulting behavior may be far from appropriate. In the Trimmer et al. model, inaction in a propitious environment is of course maladaptive with respect to immediate circumstances. Yet it arises as a byproduct of following an evolutionarily optimal decision rule and thus will not be eliminated by natural selection.

We believe that this framework will find applications beyond mood disorders. The Trimmer et al. model of depression parallels a model that we independently developed to explore why evolution has left humans vulnerable to pathological anxiety (Meacham and Bergstrom, 2016). In our model, agents are given opportunities that may be either profitable or dangerous. Before pursuing or declining each opportunity, an agent observes a cue that caries information about the likelihood of danger. Agents who are discouraged by even mild signs of threat are conceptualized as experiencing heightened anxiety, while those who ignore all but the strongest signs have reduced anxiety. In this model, an agent’s behavioral choices influence the information it has available. If the agent pursues an opportunity, it directly observes the correspondence between the signs of possible danger and the presence of an actual threat. But if the agent declines to pursue an opportunity, it does not get any information about what would have happened had it chosen to pursue. Because individuals’ experiences are idiosyncratic, we find that even when agents follow an optimal behavioral rule for modulating their sensitivity to signs of threat, some individuals will end up setting their sensitivity to threat much too high — i.e., they end up too anxious. Moreover, individuals with excess anxiety stop acquiring information and thus remain overly cautious, whereas individuals with insufficient anxiety continue acquiring information and soon correct their misperceptions. Thus, the model predicts that disorders of excess anxiety will be common but disorders of insufficient anxiety will be rare.

While these models advance our evolutionary understanding of depression and anxiety, there is plenty left to explain. Depression is not merely the expression of low mood at the wrong time; it is low mood more prolonged and more intense than what is ever seen in healthy individuals. Likewise, anxiety disorders take diverse forms, many of which look different from normal anxiety. Moreover, the negative affect of each can be so strong that sufferers are driven to attempt suicide as a means of escape. What causes these extremes so far beyond the bounds of what natural selection could favor?

One very promising avenue of investigation is to explore the role of positive feedback loops in these disorders. Feedback loops are amenable to mathematical modeling, they are common in disease generally, and they are important in mental disorders in particular. In a clinically depressed patient, low mood causes decreased motivation, which can result in poor performance at work or deteriorating social relationships, which in turn cause the sufferer to feel even more hopeless and worthless than before (Garland et al., 2002). Likewise, when a patient suffers from panic attacks, the symptoms of rising anxiety convince her that she is undergoing a health crisis and thereby further increase her anxiety (Ehlers and Margraf, 1989; Nesse and Stein, 2012). The question then becomes, how might natural selection have led to feedback loops in mood regulation that are vulnerable to dysfunction? This is a challenging problem, but we are hopeful that the mathematical modeling approach exemplified by Trimmer et al. can be productive here as well.

Another issue that is important in understanding depression is the idea that natural selection likely has not produced the best possible solution, but instead has found heuristic behavioral rules that only approach or approximate the optimal rule. Perhaps our vulnerability to depression results in part from the optimal behavioral rule being so complicated that natural selection can’t find it. This also is a difficult problem, but there is a rich history of modeling behavioral heuristcs (Hutchinson and Gigerenzer, 2005). It would be interesting to see what could be done along these lines for the problem of common mental illnesses.

In the models we have considered here, behavior arises from an evolved response strategy combined with idiosyncratic individual experience. Individual experience is determined by happenstance, but can also be influenced by behavior—creating the possibility of feedback between response strategies and the inputs that they receive. By this view, when mood or anxiety systems malfunction, they do so because of the ways that behavioral rules interact with unusual combinations of experiences and stimuli. This provides a new framework for thinking about the evolutionary vulnerability to mental disorders, a framework that incorporates both adaptive evolution and the importance of individual life experience.
